# Revisiting the Heterogeneous IFN-γ Response of Bacille of Calmette-Guérin (BCG)-Revaccinated Healthy Volunteers in a Randomized Controlled Trial: Effect of the Body Mass Index and of the *IFNG*+874 A/T Polymorphism

**DOI:** 10.1371/journal.pone.0160149

**Published:** 2016-07-29

**Authors:** Elisabete L. Conceição, Francisco S. Nascimento-Sampaio, Paulo A. Schwingel, Evelin S. Oliveira, Michael S. Rocha, Igor Vieira, Carlos M. C. Mendes, Adelmir Souza-Machado, Martha M. Oliveira, Manoel Barral-Netto, Jamocyr M. Marinho, Theolis Barbosa

**Affiliations:** 1 Universidade Federal da Bahia, Salvador, BA, Brazil; 2 Instituto Gonçalo Moniz, Fundação Oswaldo Cruz, Salvador, BA, Brazil; 3 Escola Bahiana de Medicina e Saúde Pública, Salvador, BA, Brazil; 4 Faculdade de Medicina, Universidade Federal do Rio de Janeiro, Rio de Janeiro, RJ, Brazil; 5 Hospital Santa Izabel, Salvador, BA, Brazil; Public Health England, UNITED KINGDOM

## Abstract

In trials evaluating the immune responses to Bacille of Calmette-Guérin (BCG), the genetic background and the nutritional status are host-related factors that could affect the heterogeneity in these parameters. The *IFNG*+874 A/T (rs 62559044) polymorphism has been reported to influence the IFN-γ production by BCG-vaccinated individuals challenged in vitro with mycobacterial antigens. The body mass index (BMI) is a proxy for the nutritional status and has been associated both with the susceptibility to tuberculosis and with the IFN-γ response. We show that although the *IFNG*+874 A/T polymorphism was not associated with the heterogeneity of IFN-γ production in a randomized controlled trial that evaluated long-term immune responses to BCG revaccination previously conducted in Salvador, Bahia, Brazil, the effect of this polymorphism on the observed increase in IFN-γ production among revaccinated subjects was adjusted in individuals with a low BMI.

## Introduction

The Bacille of Calmette-Guérin (BCG) vaccine has been the subject of numerous efficacy trials and epidemiological studies conducted over several decades. These trials indicate that the neonatal vaccination with BCG has 40–80% protective efficacy against tuberculosis (TB), being particularly effective against the meningeal and miliary forms of the disease, and its efficacy against the pulmonary form varies geographically [1]. New vaccines against TB are under trial [2], and the establishment of biomarkers of vaccine-induced protection could accelerate their evaluation. The study of the immune response to BCG in vaccine trials can yield important insights relative to the appropriateness of putative biomarkers of protection against TB, given the fact that BCG is the only vaccine to date proven to be protective against the disease. A putative marker related with the anti-mycobacterial response is IFN-γ. There is evidence relating the IFN-γ production with reduced disease burden in experimental disease [3] and in humans [4–6], but the in vitro IFN-γ production against mycobacterial antigen(s) and the expansion of IFN-γ-producing antigen-specific cells do not always correlate with vaccine-induced protection against TB [7,8].

The genetic background of BCG-vaccinated individuals may influence the amplitude of the post-vaccination IFN-γ response. The T>A single nucleotide polymorphism (SNP) of *IFNG* in the +874 position is one of the best studied polymorphisms that affect the IFN-γ production [9], and it has been associated both with the occurrence of active TB [10] and with lower IFN-γ production in TB patients [9] and in BCG-vaccinated children [11]. Likewise, malnutrition has been related with an increased risk of tuberculosis development [12]. A recent study conducted in Taiwan showed that body mass index (BMI) values at the beginning of the anti-tuberculosis treatment related with mortality caused by tuberculosis among male patients [13]. Also the production of pro-inflammatory cytokines including IFN-γ is impaired in *Mycobacterium tuberculosis*-infected underweight individuals [14,15].

A randomized controlled trial to evaluate the immune response to BCG revaccination was conducted in Salvador, Bahia, Brazil [16]. The volunteers enrolled in the trial were undergraduate students from two universities with a short age range, without TB infection, with high socioeconomic level, no HIV infection, no contact with tuberculosis patients and with normal complete blood counts and hematocrit. The IFN-γ response to BCG revaccination in the interval of two months after the intervention varied widely (from 0.1 to 100 fold in the revaccinated group, compared with a range of 0.1–7.1 in the control group), and was associated with the capacity of producing high levels of IFN-γ at 12-months follow-up. The study was not designed to make it possible to correlate the IFN-γ response with protection conferred by revaccination. In a previous trial conducted in the same city BCG revaccination had a modest protective effect over the neonatal BCG vaccine [17]. We discuss the possible role of *IFNG* +874 polymorphism and BMI on the observed heterogeneous IFN-γ production to in vitro stimulation with mycobacterial antigens in this trial [16].

## Materials and Methods

### Recruitment and study design

To investigate the association of the *IFNG*+874 T>A polymorphism with the IFN-γ response to *Mycobacterium tuberculosis* we compared four groups:

- Twenty-nine volunteers with distinguishable BCG vaccination scars randomly assigned to the control group in a randomized controlled trial to assess the IFN-γ response after BCG revaccination performed in Salvador, Bahia, Brazil [16].
- Forty-six volunteers with distinguishable BCG vaccination scars randomly assigned to be revaccinated in the same trial [16].
- LTBI, comprised of 66 latently infected individuals identified by routine tuberculin skin test of undergraduate health care students involved in patients’ follow-up at the Hospital Especializado Octavio Mangabeira, Salvador, Bahia, Brazil (the state reference hospital for tuberculosis diagnosis and treatment). Subjects were considered positive if they had induration above 10 mm.
- TB, comprised of 93 individuals with active pulmonary tuberculous disease, newly diagnosed by positive smear and/or culture for *Mycobacterium tuberculosis* at the Hospital Especializado Octavio Mangabeira.

This study was approved by the Ethical Committee of the Centro de Pesquisas Goncalo Moniz (CEP-CPQGM/FIOCRUZ, CAAE: 0015.0.225.000–10 and 0005.0.225.000–11), and complied with the ethical principles contained in the Brazilian National Health Council Resolution 196/96 Guidelines.

### Cultures and IFN-y production

Whole blood cultures were performed from vacuum-collected heparin treated blood, with or without mycobacterial antigen (10 μg/ml of *Mycobacterium tuberculosis* H37Rv culture lysate, Mtb, kindly provided by the Colorado State University, USA as part of NIH, NIAID Contract No. HHSN266200400091C, entitled "Tuberculosis Vaccine Testing and Research Materials", which was awarded to Colorado State University), and IFN-γ was measured in the culture supernatants as described in [16].

### Genotyping

Genomic DNA was successfully obtained for 25 revaccinated (8 male, 32%), 15 control (5 male, 33%), 66 LTBI (18 male, 27%) and 93 TB (64 male, 69%) volunteers. In this sample, height and weight values were not registered for one control (female) and five TB (3 male) participants. Height was not registered for one revaccinated (female), one LTBI (female), and 13 TB (7 male) participants. Weight was not registered for one TB (male) participant. Baseline IFN-γ production was evaluated for all revaccinated and control volunteers, as well as for 57 LTBI (among which 11 participants with values of IFN-γ in stimulated cultures that exceeded the maximum optical density of the standard curve), and 45 TB participants. IFN-γ production 2 months after intervention was available for 23 revaccinated and 8 control participants. Genomic DNA was obtained using the phenol-chloroform method. The *IFNG*+874 A/T polymorphism was investigated by Amplification Refractory Mutation System (ARMS-PCR) as previously described [18]. Briefly, to assess the presence of the A Allele, the primer IFN-γ G: 5’-TCA ACA AAG CTG ATA CTC CA-3’; and the primer IFN-γ A: 5’-TTC TTA CAA CAC AAA ATC AAA TCA-3’ were used. For the T Allele, the primer IFN-γ G was used with the primer IFN-γ T: 5’-TTC TTA CAA CAC AAA ATC AAA TCT-3’. Both reactions produce a 295-bp fragment. Amplification was performed under the following conditions: 12 μL reaction containing approximately 100 ng genomic DNA, 1μM of each primer pair, 10X buffer, MgCl2 50 mM, dNTP 25 mM and 5U/μL of Taq polymerase. The interpretation of the results was based on the presence or absence of the amplified product, which was confirmed on 2% agarose gel stained with ethidium bromide (1μg/mL).

### Statistical Analyses

The cytokine levels and ratios were compared between 2 groups using the Mann-Whitney U test for unpaired samples. All genotypes were tested for the Hardy–Weinberg equilibrium using a chi-square-test between observed and expected numbers. The Fisher’s exact test was applied to compare *IFNG* +874T/A SNP mutation frequency between the groups. The tentative Poisson model was used to evaluate the interaction among the IFN-y production and the variables genotype, sex and BMI. To assess the association among the genotypes AA and TT/TA and the ratio in the IFN-γ production (T0/T2 ≥3,3) we performed the Poisson regression. The Crude Coefficient from Poisson regression model was calculated to analyze the influence of the genotypes on the IFN-y production [19,20]. We evaluated the quality of model adjustment through Akaike information criterion (AIC), residual analysis and Variance inflation factor (Vif) [21]. The analyses were performed only in the revaccinated group, restricted to the most important variables, given the sample size. Age, body mass index (BMI [22]), and cytokine levels of the volunteers are presented as mean (95% confidence interval, CI) values. Databanks were mounted in EpiData Entry (version, EpiData Association, Denmark), and data were processed and analyzed using R (version 3.1) [23], EpiData Analysis (version) and Prism (version, GraphPad Inc., San Diego, CA).

## Results and Discussion

### Association between the IFN-γ production and the *IFNG*+874 polymorphism

**Fig 1:**
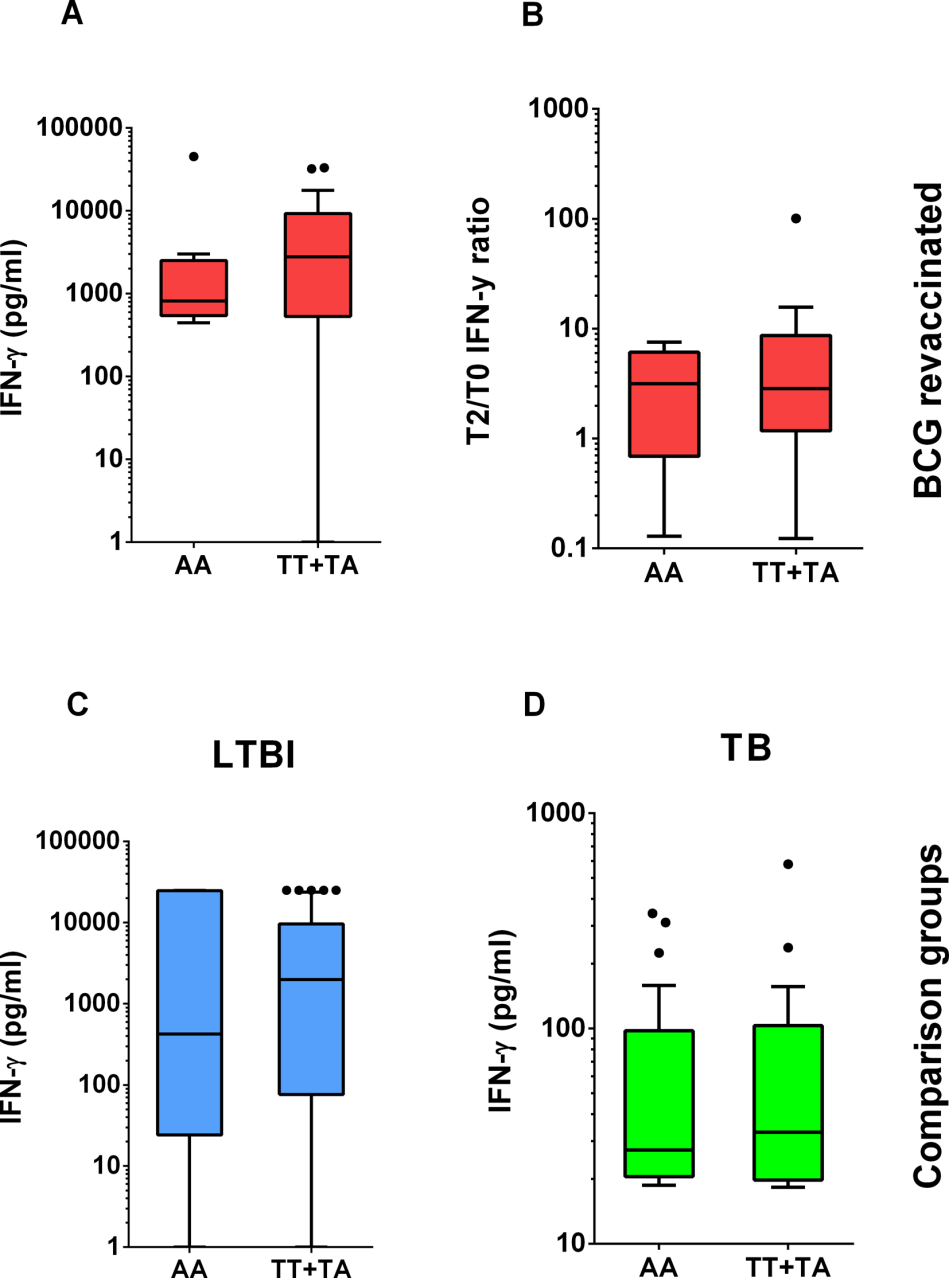
**Genotype distribution of IFN-γ production (A, C, D) or T2/T0 IFN-γ ratio (B) in whole blood cultures stimulated with mycobacterial antigen.** (A, B) BCG-revaccinated subjects; A depicts IFN-γ production at baseline. (C) LTBI volunteers. (D) TB volunteers.

The allelic and genotypic frequencies for the *IFNG*+874 polymorphism agreed with those found for the Brazilian population [18,24,25] and elsewhere [26,27]. The A allele was the most frequent and there were no differences in its frequency between the groups evaluated (Table 1). The TA genotype was the most frequent, followed by the AA genotype. The genotypic frequencies also did not differ between the groups. The individuals in all groups were stratified according to the *IFNG*+874 genotype: AA and TT/TA. The IFN-γ production at baseline (Fig 1A) and the ratio of the IFN-γ concentration measured in the 2-month follow-up culture divided by the IFN-γ concentration measured in the baseline culture (T2/T0 IFN-γ ratio) (Fig 1B) did not differ between these two strata for the revaccinated subjects. Individuals with T2/T0 IFN-γ ratio above the cut-off of 3.262 ("high-ratio") were 4.7 times more likely to produce IFN-γ above the median for the revaccinated group one year after intervention than individuals with T2/T0 IFN-γ ratio below this cut-off (“low ratio”) [16]. Furthermore, high-ratio revaccinated individuals were 7.1 times more likely to have IFN-γ production above that found for controls plus twice the standard deviation one year after the intervention [16].

**Table 1:** Distribution of genotypic and allelic frequencies of the *IFNG*+874T/A in the individuals involved in the BCG revaccination trial and in the comparison groups (LTBI and TB).

	Genotype frequency^a^			Allele frequency^b^	
	TT (%)	TA (%)	AA (%)	T (%)	A (%)
**Revaccinated (N = 25)**	2 (8)	15 (60)	8 (32)	19 (38)	31 (62)
**Controls (N = 15)**	1 (7)	10 (67)	4 (27)	12 (40)	18 (60)
**LTBI (N = 66)**	7 (11)	39 (59)	20 (30)	53 (40)	79 (60)
**TB (N = 93)**	11 (12)	47 (51)	35 (38)	69 (37)	117 (63)
**Total (N = 199)**	21 (11)	111 (56)	67 (34)	153 (38)	245 (62)

^a^Comparison between revaccinated and controls: χ2 = 0.1778, P = 1.0000; Comparison between all groups: χ2 = 2.395, P = 0.8801, AA vs TA+TT: χ2 = 1.350, P = 0.9981.

^b^Comparison between revaccinated and controls: χ2 = 0.03160, P = 1.0000; Comparison between all groups: χ2 = 0.3402, P = 0.9523.

LTBI (Fig 1C) and TB (Fig 1D) volunteers also did not present differences in IFN-γ production according to the *IFNG*+874 genotype. We speculate that polymorphisms in other positions of the *IFNG* gene probably also did not account for the heterogeneous IFN-γ response in our population because their frequency is low [28–30]. A recent study using genome-wide linkage analysis showed that the IFN-γ response of contacts of tuberculosis patients to both BCG and PPD stimulation in vitro related with the 8q11.2-8q22 region, where 108 genes are encoded including *IL7* and *LY96* [31].

### Multivariate analysis of variables influencing the heterogeneity of the IFN-γ response

**Fig 2:**
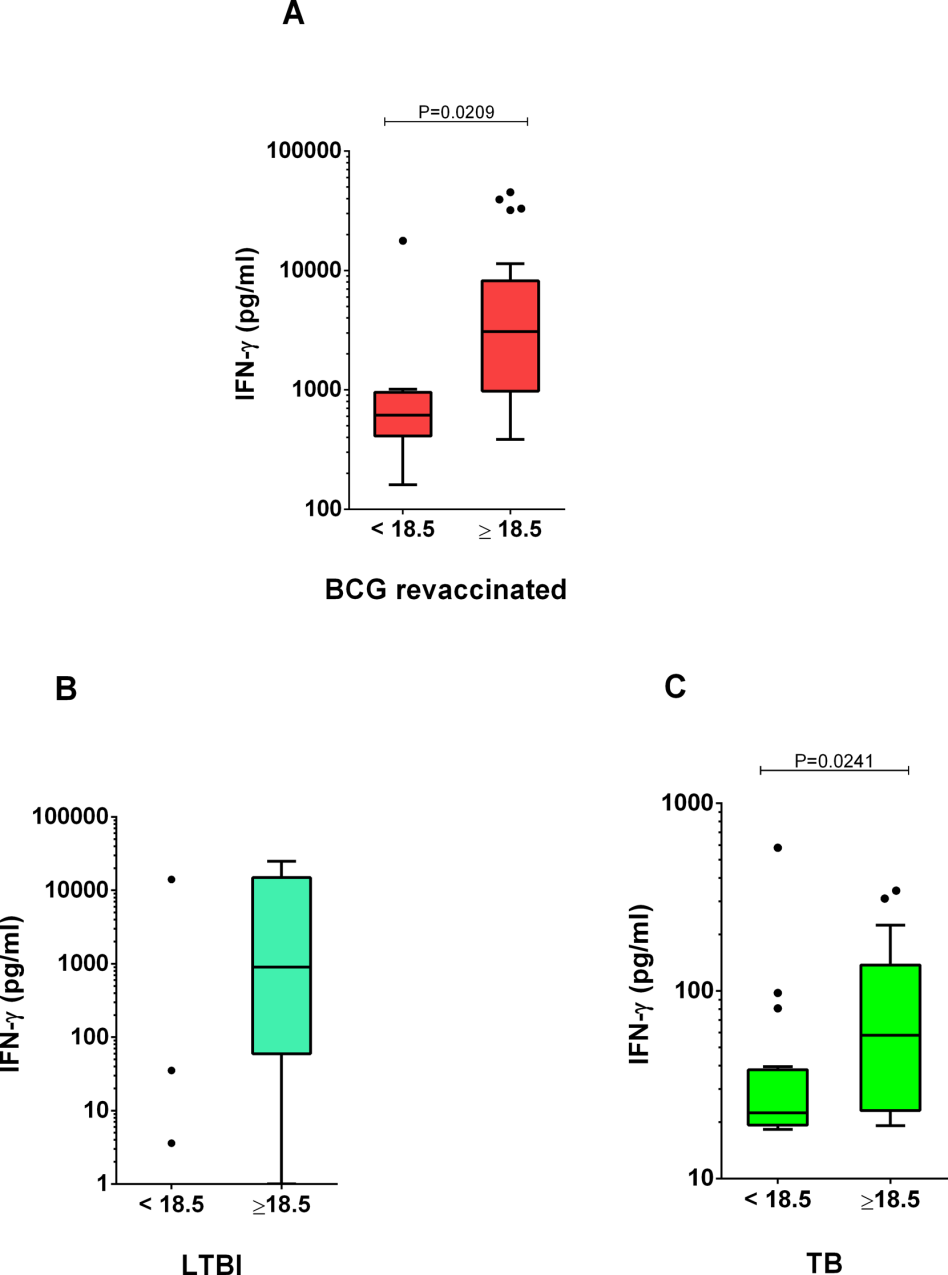
IFN-γ production in whole blood cultures stimulated with mycobacterial antigen stratified by subjects’ BMI. (A) BCG-revaccinated subjects’ baseline cultures. (B) LTBI volunteers. (C) TB volunteers. *P<0.05.

BMI did not correlate with T2/T0 IFN-γ ratio among revaccinated subjects (Spearman r = -0.0707, P = 0.7368). However, individuals in the revaccinated group with normal BMI had significantly higher IFN-γ production at baseline when compared with low BMI subjects (Fig 2A). Only three individuals with LTBI had low BMI (Fig 2B). The association between BMI and IFN-γ production was also true for the comparison group TB (Fig 2C). Therefore, we assessed the association between the T2/T0 IFN-γ ratio and the variables BMI and genotype using a multivariate model.

The results of the non-adjusted model indicated that the genotype AA increased the relative risk of belonging to the low-ratio group by 2% (Table 2). When the relative risk was adjusted by the BMI we observed that having a low BMI combined with the genotype AA decreased the relative risk of belonging to the low-ratio group by 11%. Significant interaction between a polymorphism in the beta chain of the IFN-γ receptor and BMI values influencing the risk of non-Hodgkin lymphoma among women has been reported [32]. We have not found interaction between the polymorphism in *IFNG*+874 locus and BMI (β = 0.154; RR = 1.17, P = 0.92), however we found that BMI potentially confounded the association between the *IFNG*+874 polymorphism and the T2/T0 IFN-γ ratio (Table 2) diminishing the relative risk (RR) from 1.02 to 0.89 (a moderate reduction of 12.8%). We believe it would be cautious to consider BMI when describing the association between polymorphisms in the IFN-γ axis and the IFN-γ response. We suggest that further investigation is needed to address the influence of nutrition on the effect of these polymorphisms when evaluating clinically relevant endpoints in the response to *M*. *tuberculosis*, also further dissecting pathways related with body composition [33].

**Table 2:** Multivariate analysis of the association between presenting a T2/T0 IFN-γ ratio below 3.262 (low-ratio) and the variables BMI and *IFNG*+874 genotype.

Variable	Crude Coefficient	Crude RR	P	Adjusted Coefficient	Adjusted RR	P
**(Intercepto)**	-0,5754	-	0,0840	-0,549	-	0,1300
**genotype**	0,0157		0,0979	-0,109		0,8700
**TT+TA**		1			1	
**AA**		1,02			0,89	
**BMI**		-		-0,109		0,8700
**≥ 18.5**		-			1	
**< 18.5**		-			0,89	

Note: Akaike information criterion (AIC): crude = 44,83; adjusted = 44,49. Residues analysis: mean (variance) for the crude model: -0,007 (1,05); for the adjusted model: -0,004 (1,07). Vif: absence of co-linearity between BMI and genotype.

## Conclusions

BMI and genotype may influence the IFN-γ response of individuals in tuberculosis vaccine trials, but these variables were not able to explain most of the variation found in the response to the vaccine among these subjects. It is cautious to consider BMI values in studies that address the importance of the genetic background in the immune response against mycobacteria.

## Supporting Information

S1 Dataset Individual values of IFN-γ production, *IFNG*+874 allele, sex, age, weight, height, and BMI for all participants in each group. Dots represent missing data.
(XLSX)
